# Expression of HAX-1 in colorectal cancer and its role in cancer cell growth

**DOI:** 10.3892/mmr.2015.3905

**Published:** 2015-06-11

**Authors:** XIAOLAN LI, JIANWU JIANG, RUI YANG, XIANGSHANG XU, FAYONG HU, ANDING LIU, DEDING TAO, YAN LENG, JUNBO HU, JIANPING GONG, XUELAI LUO

**Affiliations:** 1 Molecular Medicine Center, Tongji Hospital, Tongji Medical College, Huazhong University of Science and Technology, Wuhan, Hubei 430000, P.R. China; 2 Department of Gastrointestinal Surgery, The First Affiliated Hospital of Zhengzhou University, Zhengzhou, Henan 450000, P.R. China; 3 Experimental Medicine Center, Tongji Hospital, Tongji Medical College, Huazhong University of Science and Technology, Wuhan, Hubei 430000, P.R. China

**Keywords:** hematopoietic cell-specific protein 1-associated protein X-1, colorectal cancer, chemotherapy resistance

## Abstract

Colorectal cancer (CRC) is one of the most common types of cancer worldwide. Hematopoietic cell-specific protein 1-associated protein X-1 (HAX-1) has been found to be involved in several types of cancer. However, the role of HAX-1 in CRC remains to be elucidated. The aim of the present study was to investigate whether the expression of HAX-1 is associated with the progression of CRC, and to determine the effects of HAX-1 on the apoptosis and proliferation of CRC cells. Tumor tissues and adjacent noncancerous tissues were collected from 60 patients with CRC, following the provision of informed consent. The expression levels of HAX-1 and the association with clinical and pathological characteristics were then analyzed. The expression levels of HAX-1 were significantly higher in the cancerous tissues from the patients with CRC, particularly in tissues of an advanced stage of cancer. In addition, HAX-1 expression was associated with malignant progression and poor prognosis. Furthermore, SW480 CRC cells, overexpressing HAX-1, exhibited increased resistance to camptothecin *in vitro*, and promoted proliferation *in vitro* and *in vivo*. By contrast, HAX-1 knockdown significantly decreased the proliferation. In addition, the expression levels of ki-67 and phosphorylatedakt were inhibited following HAX-1 knockdown. In conclusion, the expression levels of HAX-1 were increased in cancerous tissue from patients with CRC, and were associated with progression of the disease. These results suggested that HAX-1 may contribute to chemotherapy resistance and malignant progression in CRC.

## Introduction

Colorectal cancer (CRC) is a major cause of morbidity and mortality. It is the third most common type of malignant cancer worldwide, and the fourth leading cause of cancer-associated mortality (1). The incidence of CRC is ranked only third to lung and breast cancer (2). Furthermore, it has been estimated that >143,460 new CRC cases were diagnosed in 2012, and the number of patients with CRC worldwide are expected to reach 9,000,000 by 2020 (3,4).

Despite progress in surgical and nonsurgical treatment, the prognosis for CRC remains poor; this is partially due to CRC usually being diagnosed at an advanced stage, when curative therapy is not possible. Therefore, earlier detection, diagnosis and prevention are key factors in controlling and treating CRC (5). It is well known that CRC develops as the result of a series of genetic and epigenetic alterations, which lead to the transformation of normal colorectal epithelium into colorectal adenocarcinoma (6). Identifying these key genetic alterations is critical for improving current understanding of CRC and for improving treatment strategies.

Hematopoietic cell-specific protein 1 (HS-1)-associated protein X-1 (HAX-1) was first identified as a 35 kDa protein, which interacts with HS-1, an Src kinase substrate. HAX-1 is composed of a putative transmembrane domain, a putative PEST sequence and an acid box (7). HAX-1 has emerged as an important factor in the intrinsic mitochondria-dependent pathway of cell death, which is characterized by the activation and permeabilization of mitochondria, resulting in the release of cytochrome *c* and other pro-apoptotic molecules into the cytosol (8). Previous studies have demonstrated that HAX-1 is involved in the regulation of mitochondrial membrane potential during apoptosis (9,10). In addition, HAX-1 protein interacts with a number of cellular and viral proteins (11–13). However, the function of HAX-1 remains to be fully elucidated, particularly in CRC. Several studies have demonstrated that HAX-1 may be important in apoptosis and proliferation (14,15).

In our previous study, yeast two-hybrid screening demonstrated that HAX-1 had a key role in the Salvador-warts-hippo signaling pathway, which is an important supplement for the classic apoptosis pathway (16,17). The present study aimed to analyze the potential prognostic significance of HAX-1 in CRC by detecting its expression levels in human colorectal tumor tissues, and to assess the role of HAX-1 in apoptosis and proliferation using gene-overexpression and silencing methods.

## Materials and methods

### Patients

The present study analyzed the expression levels of HAX-1 in tumor tissues from 60 patients with CRC, who were diagnosed and underwent elective surgery at the Gastrointestinal Surgery Center, Tongji Hospital (Wuhan, China) between February 2010 and November 2011. A total of 40 patients (66.7%) were >60-years old at diagnosis. The tissue samples (~2×2 cm) were collected in surgery, and were cut into three sections, one for protein extraction, one for RNA extraction and one for immunohistochemistry. The clinical stages of the CRC tissue samples were graded according to Dukes' Staging System (10). A total of 10 patients were classified as stage A, 26 patients as stage B, 14 patients as stage C and 10 patients as stage D. The current study was approved by the Ethics Committee of Tongji Hospital, Huazhong University of Science and Technology (Wuhan, China).

### Animals

Athymic female nude mice (4–6 weeks old, 15–20 g) were obtained from the Shanghai Laboratory Animal Center (Shanghai, China). Mice were fed under specific pathogen free conditions in a temperature- and humidity-controlled environment. All animal experiments were in accordance with the Institutional Animal Research Guidelines approved by the Ethics Committee. For tumor-growth studies, a total of 5×10^5^ SW48-shcon or SW480 KD1 cells were injected subcutaneously into each mouse (5 mice/group).

### Colorectal tumor tissue and cell lines

Samples of fresh tumor tissue were obtained from 60 patients with CRC and available for investigation. The tumor samples were obtained at the time of diagnosis in all cases. Institutional review board-approved, informed consent was obtained from the patients. All specimens were coded by the admission of the patient prior to analysis. The SW480 human CRC cell line and HEK293 human embryonic kidney cell line (used as a lentivirus packaging cell line) were obtained from Peking Tissue Type Culture Collection (Beijing, China). The cells were cultured in Dulbecco's modified Eagle's media (DMEM; GE Healthcare Life Sciences, Logan, UT, USA) supplemented with 10% fetal bovine serum (FBS; GE Healthcare Life Sciences) and 1% penicillin/streptomycin (Gibco Life Technologies, Carlsbad, CA, USA) at 37°C in a 5% CO_2_ incubator. (S)-(+)-CPT, G418 were purchased from Sigma-Aldrich (St. Louis, MO, USA). CPT and G418 were dissolved in dimethyl sulfoxide (Sigma-Aldrich). Cancer cells were treated with CPT (0.12 *µ*M) for 24 h and cells transfected with shRNA were screened using G418 with concentrations of 300–800 *µ*g/ml.

### Vectors and primers

Of the three empty vectors used in the present study, pEGFP-N1 (4.7 kb; cat. no. 6085-1) was purchased from Addgene (Cambridge, MA, USA); pFLAG-CMV™-4 (6.3 kb; cat. no. E7158) was purchased from Sigma-Aldrich; and pSilencer™ 2.1-U6 neo Vector (4521 bp; cat. no. 113P06) was purchased from Invitrogen Life Technologies (Carlsbad, CA, USA).

The sequences of the specific primers used for the quantitative polymerase chain reaction (qPCR) were as follows: HAX-1, sense 5′-ATGAGCCTCTTTGATCTCTTCC-3′ and antisense, 5′-CTACCGGGACCGGAACCAAC-3′ and β-actin, sense 5′-ACGTGGACATCCGCAAAGAC-3′ and antisense 5′-CTGCTGTCACCTTCACCGTTC-3′.

The HAX-1 primer sequences designed for plasmid construction were as follows: HAX-1-up-Hin*dIII*, 5′-CCAAGCTTACCATGAGCCTCTTTGATCTCTTCC-3′; and HAX-1-down-Bam*HI*, 5′-CGGGATCCCTACCGGGACC GGAACCAAC-3′. The length of fragments, amplified using qPCR with the HAX-1 and β-actin primers, were 370 and 850 bp, respectively. The primers were synthesized by Sangon Biotech Co., Ltd. (Shanghai, China).

### Sequences for short hairpin (sh)HAX-1 and small interfering (si)HAX-1

shHAX-1 and the longer lasting and more stable siHAX-1 were designed and synthesized by Guangzhou RiboBio Co., Ltd. (Guangzhou, China), as follows: shHAX-1, sense 5′-GAT CCG CGG ACA GAG ACT ACA GTA ATC AAG AGT TAC TGT AGT CTC TG TCC GTT TTT TGT CGA CGG AAA-3′ and antisense 5′-GCG CCT GTC TCT GAT GTC ATT AGT TCT CTC AAT GAC ATC AGA GAC AGG CAA AAA ACA GCT GCC TTT TCG A-3′; and shRNA-con, sense 5′-GAT CCG GTC CAT GCA TGC CGT ATC AAG AGT ACG GCA TGC ATG GAC TTT TTT GTC GAC GGA AA-3′ and antisense 5′-GCG TCC ATG CAT GCC GTA AGT TCT CTC TAC GGC ATG CAT GGA CAA AAA ACA GCT GCC TTT TCG A-3′; siHAX-1 dsRNA, sense 5′-CGG ACA GAG ACU ACA GUA A dTdT-3′ and antisense 5′-dTdT GCC UGU CUC UGA UGU CAU U-3′; scramble dsRNA, sense 5′-TCC TGT GGC ATC CAC GAA ACT-3′ and antisense 5′-GAA GCA TTT GCG GTG GAC GAT-3′. Restriction enzymes (BamHI and HindI; Santa Cruz Biotechnology, Inc., Dallas, TX, USA) were used to cut at the Bam*HI* and Hin*dIII* sites, to insert the sequences into the pSilencer™ 2.1-U6 neo Vector.

### Transfection

Transfection was performed using Lipofectamine^®^ 2000 (Invitrogen Life Technologies) in Opti-MEM (Gibco Life Technologies), according to the manufacturer's instructions. The seed density of the cells was ~30% and the final concentration of siRNA was 100 nM. The final concentrations of shRNA and the overexpression vector were 1 *µ*g/ml. The temperature of incubation was 37°C The media was replaced after 5 h.

### RNA extraction and reverse transcription-qPCR (RT-qPCR)

Subsequent to homogenization on ice with the TGrinder Electric Tissue Grinder [Tiangen Biotech (Beijing) Co., Ltd., Beijing, China], RNA was extracted from tumor tissues and the adjacent nontumor tissues and purified using RNA extraction reagent (TRIzol; Invitrogen Life Technologies), according to the manufacturer's instructions. All the RNA samples used in the present study were purified, and had optical density 260/280 ratios of 1.8–2.0 (Nanodrop Spectrophotometer; Thermo Fisher Scientific, Waltham, MA, USA). RT was performed using a Quantitect cDNA synthesis system (Qiagen, Inc., Valencia, CA, USA) in a final volume of 20 *µ*l. Actin was used as the internal control. The qPCR amplification reactions were conducted with QuantiFast SYBR Green PCRMaster Mix (Qiagen, Inc.) on the ABI 7300 Real-time PCR system (Applied Biosystems Life Technologies, Foster City, CA, USA). The reaction conductions were as follows: Enzyme activation at 95°C for 5 min, followed by 25 cycles at 94°C for 1 min, 58°C for 1 min, extension at 72°C for 1 min, and a final extension step at 72°C for 10 min. Following amplification, a melting curve was created to confirm the specificity of the reaction. The relative mRNA expression was then calculated by the 2^−ΔΔCt^ method.

### Western blot analysis

For cell lysates, cells were rinsed with ice-cold phosphate-buffered saline (PBS; Google Biotechnology Company, China), harvested, centrifuged at 4°C, and cell pellets were lysed by incubation at 4°C for 30 min in 500 *µ*l lysis buffer (Beyotime Institute of Biotechnology, Shanghai, China). For tissue lysates, tissues were homogenized with the TGrinder Electric Tissue Grinder with 500 *µ*l lysis buffer. The concentration of protein was performed using Pierce™ BCA Protein Assay kit (Thermo Fisher Scientific) and the protein quantity for each lysate was normalized to β-actin. The cell lysates were separated by SDS-PAGE (12%; Wuhan Google Biotechnology, Ltd., Wuhan, China) at room temperature, and transferred to polyvinylidene fluoride membranes (Merck Millipore, Darmstadt, Germany) on ice. The blots were probed at 4°C overnight with the following primary antibodies: Polyclonal rabbit anti-human HAX-1 (1:200; Santa Cruz Biotechnology, Inc.; sc-28268), polyclonal rabbit anti-human phosphorylated (p)-akt (S473; sc-33437) and total-akt (1:200; Santa Cruz Biotechnology, Inc.; sc-8312) and monoclonal mouse anti-β-actin (1:5,000; Sigma-Aldrich; A5316). The primary antibody was detected by chemiluminescence using horseradish peroxidase-conjugated anti-mouse (OE185739) or anti-rabbit (NL181270) immunoglobulin G secondary antibodies (Biofly Biotechnology Co., Wuhan, China).

### Immunohistochemical staining

Following deparaffinization, then rehydratation in xylene and graded (70, 98 and 100%) ethanol (Medical Company of Hubei Province, Wuhan, China), the tissue samples were incubated overnight with rabbit anti-HAX-1 (1:50, Santa Cruz Biotechnology, Inc.) diluted in 1% FBS. A GTVision™ III kit (Gene Tech Company Ltd., Shanghai, China) was used, according to the manufacturer's instructions. The sections (4–10 *µ*m thick) were then washed in water, counterstained with hematoxylin (Google Biotechnology Company, China), dehydrated in graded alcohols and xylene, and mounted onto slides using Permount (Wuhan Google Biotechnology, Ltd.). PBS was used as a blank control in place of the primary antibody. The intensity of staining was classified as 0, negative; 1, weak; 2, moderate; and 3, strong. The expression levels of HAX-1 were analyzed by classifying the immunoreactivity scores. The staining intensity was graded on a scale of 0–3 (0, no staining; 1, weak immunoreactivity; 2, moderate immunoreactivity; 3, strong immunoreactivity). The percentage of cells that exhibited positive HAX-1 staining within the normal/cancerous region of a section was scored as follows: 1, 0–25% of cells positive; 2, 26–50% positive; 3, 51–75% positive; 4, 76–100% positive for HAX-1. The staining intensity score and the percentage immunoreactivity score were then multiplied to obtain a composite score. The values of the composite score ranged from 0–12, where a score of 0–3 was defined as low expression, 4–6 was moderately high expression and >6 was high expression.

### Annexin V-fluorescein isothiocyanate (FITC)/propidium iodide (PI) and annexin V-phycoerythrin (PE)/7-aminoactinomycin D (7-AAD) detection

The SW480 cells (40–50%) were resuspended in 500 *µ*l 1X binding buffer for 12 h at 37°C in 5% CO_2_; subsequently 5 *µ*l Annexin V and 5 *µ*l PI/7-AAD (all from BD Biosciences, Franklin Lakes, NJ, USA) were added to the binding buffer. The cells were then gently suspended and incubated for 15–20 min at room temperature in the dark, prior to being analyzed using flow cytometry (FACSVantage; BD Biosciences) (18).

### Bromodeoxyuridine (BrdU) assay

A total of 10 *µ*M BrdU (Sigma-Aldrich) was added to the cells 1–2 h prior to harvesting. The cells were then fixed in 70–80% cold ethanol and maintained at −20°C overnight. The fixed cells were resuspended, following being washed in 1 ml 4 M HCl (Google Biotechnology Company, China) at room temperature for 30–50 min in 0.1 M Brox solution (Sigma-Aldrich). Monoclonal anti-BrdU antibody (1:200; BioLegend, San Diego, CA, USA) was then added, and the cells were incubated in the dark at 4°C for ≥1 h. The cells were then rinsed and incubated with a secondary FITC-conjugated antibody (Dako, Glostrup, Denmark) for 30 min in the dark at 4°C. After being incubated with 500 *µ*l solution containing 50 *µ*g/ml PI and 50 *µ*g/ml RNase at room temperature for 20–30 min in the dark, the cells were analyzed by flow cytometry.

### Carboxyfluorescein succinimidyl ester (CFSE) proliferation assay

The harvested cells were washed twice in cold PBS and then suspended in 10 ml labeling reaction dilution (Sigma-Aldrich). Following the addition of 200 *µ*l 0.5 mM CFSE (Beyotime Institute of Biotechnology, Haimen, China), according to the manufacturer's instructions, the cells were incubated at 37°C for 10 min. The reaction was terminated by adding 10 ml 10% FBS complete DMEM media, and the cells were washed twice. Subsequently, the cells were cultured at 37°C for 0, 16, 24, 36 and 48 h, and analyzed using flow cytometry. The proliferation index was calculated using ModFit LT for Mac V2.0 (BD Biosciences).

### Statistical analysis

The t-test and c-test (or Fisher's exact test) were conducted using SPSS software, version 19.0 (IBM SPSS, Armonk, NY, USA) software. P<0.05 was considered to indicate a statistically significant difference.

## Results

### Expression of HAX-1 and correlation with the prognosis of CRC

**Figure 1:**
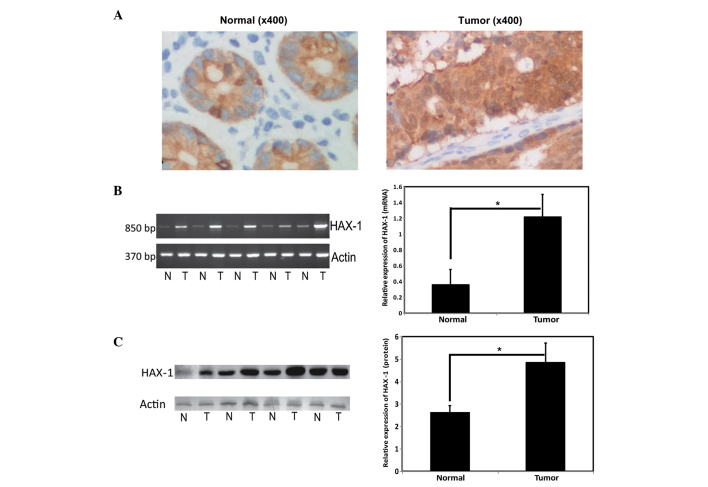
Expression of HAX-1 in CRC tissue. (A) Representative immunohistochemical images of CRC tissue (magnification, x400), Left, normal tissue; right, tumor tissue). The expression levels of HAX-1 in CRC tissues were observed to be significantly higher than those in benign/adjacent noncancerous tissues. (B) mRNA expression levels of HAX-1 in colorectal cancer tissues (Left, reverse transcription-quantitative polymerase chain reaction; right, normalized with actin.) ^*^P<0.05. Values are presented as the mean ± standard deviation. (C) Protein expression levels of HAX-1 in CRC tissues (Left, western blotting; right, normalized with acctin). ^*^P<0.05. Values are presented as the mean ± standard deviation. HAX-1, hematopoietic cell specific protein 1-associated protein X-1; N, normal tissue; T, tumor tissue. CRC, colorectal cancer.

The expression levels of HAX-1 in the individual tumor and noncancerous tissue sections from the 60 patients with CRC were detected using immunohistochemistry. As shown in Fig. 1, HAX-1 was ubiquitously expressed in the colorectal tissues, as reported previously (19,20). Notably, the most prominent expression of HAX-1 was detected in the mucosal membrane of the colon, and was predominantly located in the cytoplasm (Fig. 1A). The mRNA and protein expression levels of HAX-1 were detected using RT-qPCR and western blotting, respectively. The mRNA and protein expression levels of HAX-1 were significantly higher in the CRC tissues, than in the benign tissues (Fig. 1B and C).

Correlations between the expression levels of HAX-1 and the clinical data obtained from the patients are summarized in Table I. The clinical factors evaluated included age, gender, differentiation, lymph node metastasis, muscular layer invasion, pathology type and Dukes' stage. In addition, the patients were stratified into high and moderate high groups according to the expression of HAX-1. Of the 60 primary CRC tissue samples, 50 exhibited high expression levels of HAX-1. The high rate of expression was significantly increased in the patients with CRC with lymph node metastasis, compared with those without lymph node metastasis (91.67, vs. 75.00%, respectively). Furthermore, patients with muscular layer invasion exhibited higher expression levels of HAX-1, compared with those without muscular layer invasion (90.2, vs. 44.44%, respectively). A total of 22 of the 24 patients (91.67%) with Dukes C+D stages also exhibited high expression levels of HAX-1. The expression levels of HAX-1 were also increased in 24 of the patients with Dukes A+B; however, the rate of increased expression was only 75.00%. No significant correlations were observed between the expression of HAX-1 and other prognostic factors, including, age, differentiation and pathology type.

### Effects of the expression of HAX-1 on cell apoptosis

**Figure 2:**
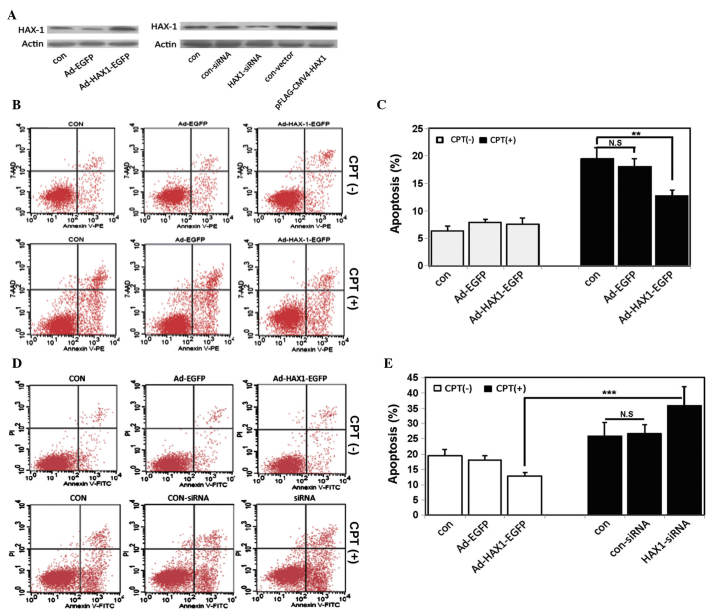
Effects of the expression of HAX-1 on apoptosis. (A) SW480 human colorectal cancer cells were transfected with an HAX-1 overexpression vector (upper panel) or an HAX-1-siRNA vector (lower panel) for 48 h. The protein expression levels of HAX-1 were detected using western blotting. (B) Representative annexin V/7-AAD dot-plot distributions of SW480 cells treated without (upper panel) or with (lower panel) CPT in the different groups: Con, SW480 cells without infection; Ad-EGFP, SW480 cells infected with empty vector; Ad-HAX-1-EGFP, SW480 cells infected with Ad-HAX-1-EGFP vector. (C) Quantification of apoptosis. Experiments were repeated at least three times. Values represent the mean ± standard deviation (^**^P<0.01). (D) Representative annexin V/PI dot-plot distributions of transfected SW480 cells treated without (upper panel) or with (lower panel) CPT in the different groups: Con, SW480 cells without transfection; con-siRNA, SW480 cells transfected with control siRNA; siRNA, SW480 cells transfected with HAX-1-siRNA. (E) Quantification of apoptosis. Experiments were repeated at least three times. Values represent the mean ± standard deviation (^***^P<0.001). HAX-1, hematopoietic cell-specific protein 1-associated protein X-1; siRNA, small interfering RNA; CPT, camptothecin; siRNA, small interfering RNA; N.S, not significant.

To identify the effects of HAX-1 on apoptosis, HAX-1-overexpressing SW480 cells were constructed by transfecting the cells with an adenovirus vector containing the EGFP expressing reporter (Ad-HAX-1-EGFP). Western blot analysis was performed 48 h post-transfection, which revealed that HAX-1 was overexpressed in the SW480 cells transfected with the Ad-HAX-1-EGFP plasmid (Fig. 2A, upper panel). A significant increase in the resistance of the HAX-1-transfected SW480 cells to camptothecin (CPT) was observed 8 h following administration, whereas the SW480 cells without HAX-1 (Ad-EGFP) did not exhibit resistance to CPT (Fig. 2B and C). In addition, apoptosis was reduced in the SW480 cells overexpressing-HAX-1.

To further confirm the effects of HAX-1 on apoptosis, the expression of HAX-1 was silenced using an HAX-1 siRNA plasmid. As shown in Fig. 2, the mRNA and protein expression levels of HAX-1 were significantly decreased in the SW480 cells following transfection with HAX-1 siRNA (Fig. 2A, lower panel). Following treatment with CPT for 8 h, apoptosis was increased in the SW480 cells transfected with the HAX-1 siRNA plasmid (Fig. 2D and E). These results were consistent with those of a previous study, which demonstrated that HAX-1 had a functional role in inhibiting apoptosis in CRC (7,21).

### Effects of HAX-1 expression on cell proliferation

**Figure 3:**
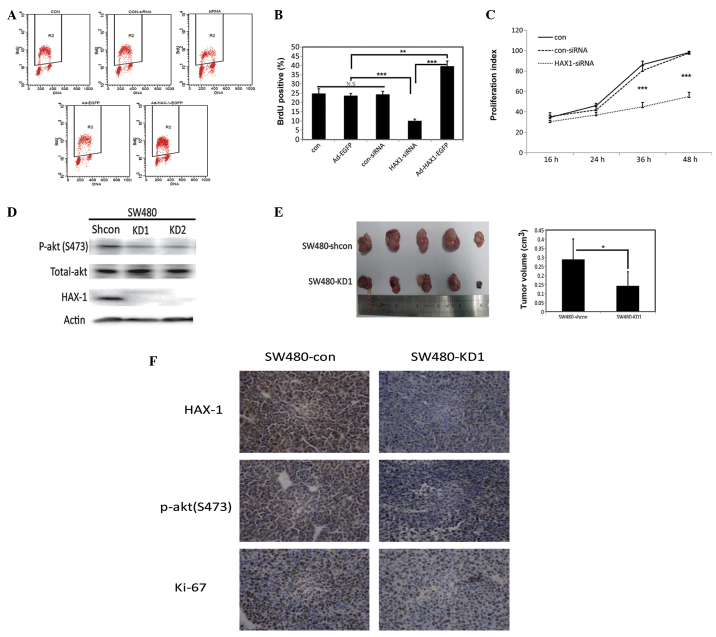
Effects of the expression of HAX-1 on proliferation. (A) Representative dot-plot distributions of BrdU assay using flow cytometry in the SW480 human colorectal cancer cells transfected with Ad-EGFP, Ad-HAX-1-EGFP, con-siRNA or HAX-1-siRNA for 48 h. (Con, SW480 cells without infection; Ad-EGFP, SW480 cells infected with empty vector; Ad-HAX-1-EGFP, SW480 cells infected with Ad-HAX-1-EGFP vector; con-siRNA, SW480 cells transfected with control siRNA; siRNA, SW480 cells transfected with HAX-1-siRNA). The R2, gate represents the percentage of BrdU positive cells. (B) Quantification of Brdu positive cells. Experiments were repeated at least three times. Values represent the mean ± standard deviation (^**^P<0.01; ^***^P<0.001). (C) Proliferation index curve of SW480 cells transfected with con-siRNA and HAX-1-siRNA for 48 h. The proliferation index was calculated based on fluorescence intensity, detected using a CFSE assay. (Con, SW480 cells without transfection; con-siRNA, SW480 cells transfected with scramble siRNA; HAX-1-siRNA, SW480 cells transfected with HAX-1-siRNA). (D) Western blot analysis of the expression levels of p-akt (S473), total-akt and HAX-1 in SW480 cells transfected with shRNA-con (Shcon), shRNA1 (KD1) or shRNA2 (KD2) vectors for 48 h. (E) Left panel shows xenografts of BALB/c mice (n=5) transplanted with 5×10^5^ HAX-1-knockdown SW480 cells (SW480-KD1), compared with the control (SW480-shcon). The right panel shows quantification of tumor volumes Values are presented as the mean ± standard deviation (^*^P<0.05). (F) Representative immunohistochemical images of HAX-1, p-akt (S473) and Ki-67 in the mice xenografts. HAX-1, hematopoietic cell-specific protein 1-associated protein X-1; shRNA, short hairpin RNA; siRNA, small interfering RNA; BrdU, bromodeoxyuridine; siRNA, small interfering RNA; shRNA, short hairpin; N.S, not significant.

CRC cells present with malignant proliferative properties. To investigate the effects of HAX-1 on CRC cell proliferation, a Brdu incorporation assay for DNA synthesis was performed. The overexpression of HAX-1 promoted DNA synthesis, as indicated by the increased number of Brdu-postive cells. However, this promotion was repressed when the expression of HAX-1 was silenced by siRNA, compared with the control SW480 cells (Figs. 3A and B). Similar results were observed in the CFSE assay (Fig. 3C).

The proliferation of SW480 cells was markedly decreased when the cells were transiently transfected with anti-HAX-1 siRNA for 48 h. To further understand the effects of HAX-1 on cell proliferation, shRNA-HAX-1 was constructed. Stable HAX-1-knockdown SW480 cell lines were generated through transfection of the cells with potent shRNA targeting HAX-1 (SW480-KD), and the cells were subsequently screened using G418. The knockdown efficiencies were then measured using western blotting (Fig. 3D). Two stable clones, SW480-KD1 and SW480-KD2, were obtained. A total of 5×10^5^ SW480-KD1 cells were then subcutaneously implanted into nude mice, and primary tumor growth was monitored. SW480-shcon cells were injected into the control mice at the same time. After 2 weeks, the tumors were dissected and sizes of the tumor masses were measured. As expected, the size of the tumor masses from the HAX-1-knockdown mice were significantly smaller compared with those fron the mice in the control group (Fig. 3E). Immunohistochemical analysis of the xenografts from the mice injected with the SW480-KD1 cells revealed a decrease in the expression of ki-67, indicating that proliferation was markedly inhibited (Fig. 3F). As shown in Fig. 3D and F, the protein expression levels of p-akt (S473) were decreased in the xenografts from the mice injected with the SW480-KD1 cells. These results suggested that HAX-1 may be involved in the AKT signaling pathway.

## Discussion

HAX-1 is a multifunctional protein, which has been recognized as a regulator of apoptosis and cell survival (7,8,13,22). The expression of HAX-1 is increased in numerous malignancies, including lung cancer, breast cancer, CRC, lymphoma, melanoma and leukemia (23–26). In the present study, the expression of HAX-1 in the CRC tissues was determined using immunohistochemistry, RT-qPCR and western blotting. The results of the present study demonstrated that the expression levels of HAX-1 were significantly upregulated in the tumor specimens, compared with the adjacent noncancerous tissues, which was concordant with the findings of previous studies (25,27). Previous reports have demonstrated that patients with esophageal squamous cell carcinoma overexpressing HAX-1 are more likely to exhibit lymph node metastasis and have a poor prognosis (27). It has also been demonstrated that upregulated HAX-1 is associated with advanced clinicopathological characteristics and prognosis in CRC (25). The present study demonstrated that the expression of HAX-1 was significantly associated with lymph node metastasis, muscular layer invasion and clinical stage in CRC, which suggested that HAX-1 may be important in the progression and metastasis of CRC.

Apoptosis is involved in the regulation of cell survival and homeostasis, and abnormal apoptotic processes can promote carcinogenesis and chemotherapy resistance (28,29). Previous studies have reported on HAX-1-mediated chemotherapy resistance in T-cell leukemia and melanoma (26,30). In the present study, ectopic overexpression of HAX-1 protected SW480 cells from CPT, an agent that induces apoptosis via activation of the intrinsic mitochondrial pathway (31). Conversely, the protection is decreased when HAX-1 is suppressed by siRNA. These results support the hypothesis that high expression levels of HAX-1 protect CRC cells from genotoxic agents.

The results of the present study indicated that HAX-1 was involved in the proliferation of CRC. Cellular proliferation was monitored using BrdU and CFSE assays, which demonstrated that upregulating the expression of HAX-1 promoted DNA synthesis. However, this proliferation was inhibited by HAX-1-siRNA. To confirm this finding, stable HAX-1-knockdown (SW480-KD) and control (SW480-shcon) cells were implanted into nude mice. The results revealed that downregulating the expression of HAX-1 in the SW480 cells suppressed tumor growth *in vivo* in the mice xenograft model. These results suggested that HAX-1 may be a potent therapeutic molecular target for inhibiting the growth of human cancer.

In conclusion, the present study demonstrated that the expression levels of HAX-1 were significantly increased in colorectal tissues, and may be important in the apoptosis and proliferation of CRC cells. Further investigations are required to elucidate the mechanism underlying the effects of HAX-1 on cell apoptosis and proliferation.

## Figures and Tables

**Table I:** Association between the gene expression levels of HAX-1 and prognostic factors in 60 patients with colorectal cancer.

Clinical factor	Number of patients (n)	Expression of HAX-1 (n)		High expression rate (%)	P-value
		High	Moderate high		
Age (years)					>0.05
<60	20	17	3	85.00	
>60	40	33	7	82.50	
Gender					>0.05
Male	41	35	6	85.37	
Female	19	15	4	78.95	
Differentiation					>0.05
Low	9	9	0	100	
High/Moderate	51	41	10	80.39	
Lymph node metastasis					0.025
No	36	27	9	75.00	
Yes	24	22	2	91.67	
Muscular layer invasion					0.001
No	9	4	5	44.44	
Yes	51	46	5	90.20	
Pathology type					>0.05
Adenocarcinoma	53	44	9	83.02	
Mucinous adenocarcinoma	7	5	2	71.43	
Dukes stage					0.025
A+B	36	27	9	75.00	
C+D	24	22	2	91.67	

Fisher's exact test was used to determine significance. HAX-1, hematopoietic cell-specific protein 1-associated protein X-1.
